# Novel technical modifications of hand-assisted laparoscopic dissection of a large obturator mass: A case report and literature review

**DOI:** 10.3892/ol.2021.12834

**Published:** 2021-06-02

**Authors:** Jun Zhang, Peng Zhong, Haipeng Tong, Jun Jiang, Dali Tong

Oncol Lett 20: Article no. 269, 2020; DOI: 10.3892/ol.2020.12132

Following the publication of the above article, the authors have realized that the grant number in the Funding section of the Declarations at the top of p. 6 for the support provided by the Dali Tong Chongqing Science and Technology Bureau (Basic and Frontier Research Project) was written incorrectly;

essentially, ‘cstc2018jcyjA2133’ was the acceptance number, and the project number (cstc2018jcyjAX0645) should have been written here instead.

Therefore, the text for the Funding section should have been written as follows (the changed text is highlighted in bold): “This work was supported in part by the Dali Tong Chongqing Science and Technology Bureau (Basic and Frontier Research Project; grant no. **cstc2018jcyjAX0645**) and the Chongqing Municipal Health and Health Committee (Science and Health Joint Medical Research Project; grant no. 2018QNXM041).”

The authors regret their oversight in providing this incorrect information in the Funding section of their paper. They thank the Editor of *Oncology Letters* for allowing them the opportunity to publish this corrigendum, and apologize to the readership of the Journal and to the inappropriately credited funding body in question for any inconvenience caused.

